# Evaluation of Antibacterial, Antifungal, Antiviral, and Anticancer Potential of Extract from the Fern *Dryopteris erythrosora*

**DOI:** 10.3390/ijms26115182

**Published:** 2025-05-28

**Authors:** Kamila Górka, Marcin Koleśnik, Kinga Salwa, Mateusz Kwaśnik, Konrad Kubiński

**Affiliations:** 1 Department of Molecular Biology, The John Paul II Catholic University of Lublin, Konstantynów 1 I, 20-708 Lublin, Poland; mateusz.kwasnik@kul.pl; 2 Department of Virology with Viral Diagnostics Laboratory, Medical University of Lublin, Chodźki 1, 20-093 Lublin, Poland; marcin.kolesnik@umlub.pl (M.K.); kinga.salwa@umlub.pl (K.S.)

**Keywords:** *Dryopteris erythrosora*, plant extract, antimicrobial activity, anticancer activity, antiviral activity, cytotoxicity

## Abstract

Plant extracts are increasingly being investigated due to their high content of pharmacologically active substances. The primary focus is placed on angiosperms, while pteridophytes are less popular, although their medicinal properties have been recognized for centuries. In this study, we uncover some biological properties of the extract from *Dryopteris erythrosora* (DEE), a fern traditionally used for liver treatment in Asia, which has not been widely explored in this context before. This study involved the determination of the total content of polyphenols and flavonoids as well as the evaluation of the antioxidant potential of DEE. Its antimicrobial activity was tested against selected bacteria. The MIC values ranged from 1.25 to 0.375 mg/mL. DEE showed no inhibitory effect against a representative fungus, *Candida albicans*. Additionally, this study demonstrated its excellent anticancer activity against AGS, MCF-7, and SW-480 cancer cells, with IC_50_ values of 19.44, 76.90, and 24.97 μg/mL, respectively. A study on human herpesvirus type 1 (HHV-1) revealed that the DEE had no antiviral activity. The safety of DEE was confirmed with the use of sheep erythrocytes and VERO cells. Since *D. erythrosora* is a rich source of compounds with antibacterial and anticancer properties, it can complement the arsenal of natural therapeutics.

## 1. Introduction

Recently, the use of plants and their extracts as rich sources of natural chemical compounds with pharmacological properties has become widespread. Therapy based on natural products derived from plants is gaining increasing attention, as—unlike many conventional treatments—it is generally associated with fewer adverse side effects. According to available data, approximately 25–50% of all drugs in clinical use are of plant origin [1,2,3]. The World Health Organization (WHO) reports that approximately 11% of the 252 drugs classified as essential medicines are exclusively derived from plants, while a significant proportion of the remaining drugs are synthesized using natural precursors. Estimates suggest that more than half of the currently used anticancer and anti-infective drugs are of natural origin. These data clearly highlight the crucial role of bioactive plant-derived compounds in modern medicine [1,2,3]. One example of a medicinal plant is *Withania somnifera*, commonly known as Ashwagandha, which is well known for its anticancer properties. In addition, it exhibits anxiolytic, antirheumatic, and anti-inflammatory effects. Another example is the extract of *Achyranthes aspera*, which has shown anticancer, antioxidant, hepatoprotective, diuretic, and wound-healing properties [4]. *Centaurium erythraea* is a plant with a wide range of therapeutic effects. It is used in the treatment of diabetes, digestive disorders, pneumonia, and cardiovascular diseases [5]. Studies have shown that *Verbascum thapsus* L. possesses antiviral, analgesic, sedative, antibacterial, and antifungal properties [6]. The extract of *Verbena officinalis* is also widely used in traditional medicine, primarily due to its anti-inflammatory, neuroprotective, analgesic, and anticonvulsant effects [7].

Many studies indicate the promising role of phytochemicals naturally contained in plants. Their main sources used by researchers are mainly angiosperms due to their widespread distribution and high biological diversity. Pteridophytes are much less popular, even though they are also used in traditional medicine and their healing properties have been known for centuries [8]. Ferns are one of the oldest groups of vascular plants found around the world [8,9]. They were the basis for hepatitis treatment in China, where many ferns are native species, including *Dryopteris erythrosora*, which also occurs naturally in Japan, where its popular name “Japanese red” comes from. This is related to the fact that young leaves of this plant are reddish; as the plant develops, they turn green, but in the fall, they have an orange–red shade again. The main components of pteridophytes that are believed to have healing properties are flavonoids and terpenoids. These groups of compounds are characterized by antioxidant properties playing an important role in anti-inflammatory, anticancer, antigenotoxic, and protective effects in neurodegenerative diseases [10].

**Figure 1:**
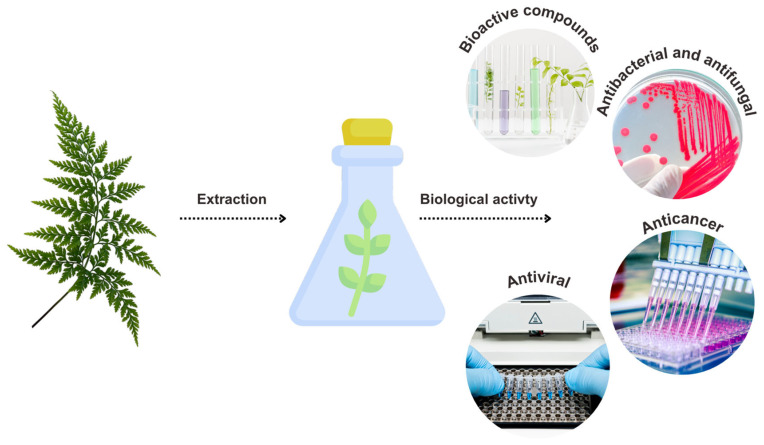
Schematic overview of the experimental design for evaluating the biological activity of the *Dryopteris erythrosora* extract (DEE) presented in this study.

Currently, there are only several reports on *D. erythrosora* in the literature. A search of a popular database has shown only several scientific articles referring to the record “*Dryopteris erythrosora*”. In the PubMed database, 12 records have been found since 2005. In the ScienceDirect database, there are 66 articles, although only 4 of them are related to the biological activity of *D. erythrosora*. In turn, no records were found with *Dryopteris erythrosora* in the title in the Wiley Online Library. Most studies are only focused on the determination of the content of flavonoids in various parts of the plant. The only data on the biological activity of extracts from *D. erythrosora* that we found refer to a flavonoid extract that showed anticancer activity against the A549 cell line [10] and to the antibacterial activity of a *D. erythrosora* methanolic extract, which was active against *Staphylococcus aureus* [11]. To the best of our knowledge, there is no other information on the biological activity of this plant. Therefore, the aim of our study was to evaluate, for the first time, its antifungal and antibacterial activity against other microbial species. Moreover, we examined the anticancer and antiviral potential of DEE as well as its safety for normal cells (Figure 1).

The conducted research may contribute to expanding the current knowledge on the biological activity of ferns and support the discovery of new drugs or adjuvant therapies, which is particularly valuable in the context of the increasing microbial resistance to antibiotics and the limited effectiveness of many chemotherapeutic agents. Furthermore, the present results may serve as a basis for future studies on the potential application of *D. erythrosora* in phytotherapy, dietary supplements, cosmetics, and nutraceuticals.

## 2. Results and Discussion

### 2.1. Bioactive Compounds and Antioxidant Potential of DEE

Since plants are a rich source of a plethora of bioactive molecules and the fern of interest has not been comprehensively studied in this respect, the aim of this study was to determine selected biological activities of ethanolic *D. erythrosora* extract (DEE). In order to determine the total phenolic content and the total flavonoid content in DEE, standard curves of absorbance versus the concentration of gallic acid and quercetin, respectively, were plotted. The total phenolic content (TPC) expressed as gallic acid equivalent and the total flavonoid content (TFC) expressed as quercetin equivalent were calculated. Per 1 g of dry extract, the total flavonoid content (TFC) was 16.1 mg quercetin equivalent (QE), while the total phenolic content (TPC) was 36.6 mg gallic acid equivalent (GAE).

**Figure 2:**
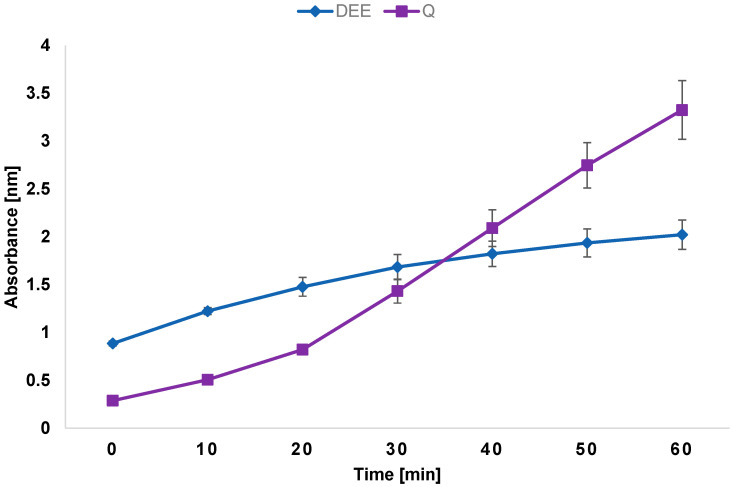
Antioxidant power of 1 mg/mL DEE and 1 mg/mL quercetin assessed by the FRAP assay.

Polyphenols, mainly flavonoids, are responsible for the antioxidant properties of plant extracts, which are very desirable, because many pathological states are related to oxidative stress [12]. The reduction potential of DEE was tested using the FRAP (Ferric Reducing Antioxidant Power) assay. The assay measures the antioxidant potential at which Fe^3+^ is reduced by an antioxidant to Fe^2+^. The resulting Fe^2+^ forms a specific color complex with a chromogen. The color intensity at 590 nm is proportional to the FRAP in the sample. Figure 2 shows the reducing antioxidant power of DEE assessed using the FRAP assay. Quercetin was used as a reference standard. As shown in Figure 2, the FRAP of DEE and quercetin increased with the increasing time.

**Figure 3:**
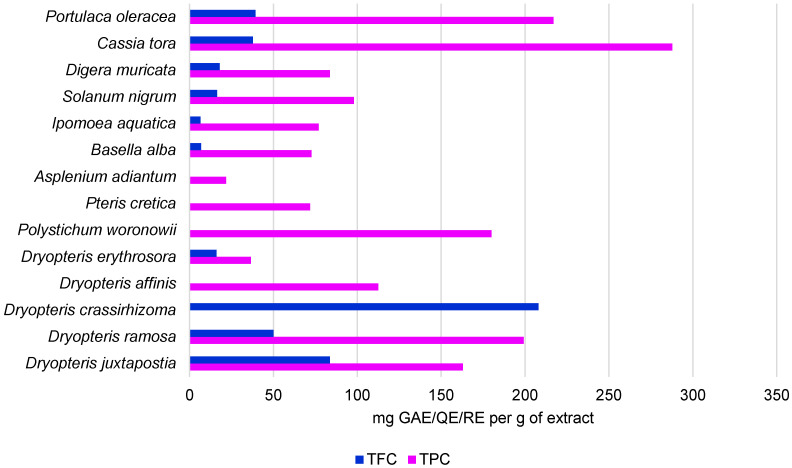
Total content of polyphenols and flavonoids in plants of the genus *Dryopteris* and in other ferns and higher plants [18,20,21,22,23].

The results show that, in contrast to other higher plants, the content of polyphenols and flavonoids in *D. erythrosora* appears to be rather low [13,14,15,16,17]. The content of polyphenols and flavonoids was determined in another Dryopteris species, i.e., *Dryopteris juxtapostia*. In a study conducted by Rani et al. [18], the content of phytochemical components was examined in methanolic and dichloromethane (DCM) extracts from the roots and shoots of the fern. The highest polyphenol content was found in the DCM root extract and the lowest amounts were detected in the methanolic shoot extract, i.e., 222 ± 0.41 mg GAE/g and 91.4 ± 0.2 mg GAE/g, respectively. However, the total flavonoid content recorded in the extracts ranged between 83.7 ± 0.1 mg QE/g and 13.2 ± 0.5 mg QE/g, depending on the part of the plant and the solvent. Comparing the results obtained here to methanolic extracts from angiosperm plants, the total flavonoid content was in our case approximately 2 times higher (*Ipomoea aquatica*, *Basella alba*), similar (*Solanum nigrum*, *Digera muricata*), or approximately 3 times lower (*Cassia tora*, *Portulaca oleracea*), depending on the species [19]. Considering the content of polyphenols and flavonoids in different higher plants reported in many other studies, the present results appear to be significantly different (Table 1, Figure 3). However, this is probably related to the use of different solvents, which means that other bioactive compounds may have been present in the tested extracts. Therefore, it is necessary to further determine the phytochemical composition of *D. erythrosora* using different solvents to observe possible differences.

In a study reported by Cao et al. [10], flavonoids present in *D. erythrosora* were identified using LC-DAD-ESI/MS. The total flavonoid content was determined to be 14.33%. The main flavonoids in *D. erythrosora* were identified as flavonol glycosides. The following compounds were detected in the extract: gliricidin 7-O-hexoside, monogalloyl-glucose, apigenin7-O-glucoside, quercetin 7-O-rutinoside, quercetin 7-O-galactoside, quercetin, myricetin 3-O-rhamnoside, kaempferol 7-O-gentiobioside, kaempferol-3-O-rutinoside, and others. In their HPLC analysis of extracts from various parts of *D. erythrosora*, Zhang et al. [24] identified such flavonoids as dihydromyricetin, luteolin, baicalin, catechin, wogonin, and kaempferide. The total flavonoid content in *D. erythrosora* leaves, stems, rachis, and roots determined in their study ranged from 2.1% to 8.26%. Chang et al. [25] reported that the total flavonoid content in *D. erythrosora* leaves was around 0.89%. This difference may result from differences in the extraction conditions and from the fact that, in their study, the dry extract was dissolved in different solvents before the test.

With regard to the reducing antioxidant power of 1 mg/mL DEE, the result was approximately 3 times lower than that of 1 mg/mL quercetin. Quercetin is a compound with strong reducing properties and is therefore commonly used as a reference substance in antioxidant activity assays. It is capable of rapidly reducing the Fe^3+^–TPTZ (2,4,6-Tris(2-pyridyl)-s-triazine) complex to Fe^2+^, resulting in a quick and pronounced increase in absorbance. The results show that the extract did not induce such a rapid or distinct increase in absorbance, which suggests that it has a weaker reducing power than quercetin. However, it should be noted that the tested extract is a mixture of compounds. In addition to antioxidant constituents, it also contains other substances, such as proteins and sugars, which may interfere with the course of the reaction. Therefore, future studies should focus on the identification and isolation of active compounds present in the extract, followed by an evaluation of their individual antioxidant potential. Nevertheless, this test represents a preliminary analysis that still demonstrates that DEE exhibits antioxidant activity. The present results correspond to those reported by Cao et al. [10], where the antioxidant power of the flavonoid extract from *D. erythrosora* was weaker than that induced by rutin.

### 2.2. Antimicrobial Potential of DEE

Since many plant-derived polyphenols show antimicrobial potential, such properties of the DEE were determined against *Escherichia coli*, *Klebsiella pneumoniae*, *Staphylococcus aureus*, *Staphylococcus epidermidis*, and *Candida albicans* using the broth microdilution method with calculation of the minimum inhibitory concentration (MIC). DEE was tested at concentrations ranging from 0.023 to 10 mg/mL. The results showed that the extracts at the given concentrations did not affect the growth of the fungus *C. albicans*. The tested bacteria were sensitive to the extracts, with MIC values ranging from 0.375 to 1.25 mg/mL (Table 2). The *S. aureus* strain turned out to be the most sensitive, while *E. coli* was the most resistant.

The present results are slightly higher than those reported by Yun and Bai [11], who examined the effect of *D. erythrosora* extract on *S. aureus* strains. They obtained MIC values in the range of 0.125 and 0.250 mg/mL. Another species of Dryopteris whose antibacterial effect was tested against *S. aureus* was *D. crassirhizoma*. The methanol extract of this plant showed activity with a MIC value of 31.25 mg/mL, which is 80 times higher than the MIC against *S. aureus* determined in this study for *D. erythrosora* extract [26]. Another example of a fern that can be a source of antimicrobial activity is *Woodwardia unigemmata*, with MICs of 10 mg/mL against *L. monocytogenes* [27]. In summary, based on the referenced studies on the antibacterial activity of ferns and the classifications of antimicrobial activity of plant extracts presented in Table 3, it can be concluded that DEE showed promising efficacy.

Bussmann et al. [30] conducted a study on the antimicrobial activity of 141 species of medicinal plants traditionally used in northern Peru. Plant extracts were prepared similarly to the DEE. The aerial parts of the plants used for the study were dried and ground using a mill. The samples were then extracted with 96% ethanol. After evaporation of the solvent, the dry extract was dissolved in water and subjected to further testing. The MIC value was determined using the broth microdilution method against *S*. *aureus* and *E*. *coli*. In most cases, the MIC values were relatively high, ranging from 0.008 to 256 mg/mL. Selected results of the antimicrobial activity of the tested plants are presented in Table 4. Based on the cited results, it can also be concluded that DEE exhibits good antibacterial activity against *S. aureus* and *E. coli*.

In comparison to other higher plants, the antimicrobial potential of DEE seems to be moderate. In a study conducted by Kang et al. [31], a methanolic extract from 12 higher medicinal plants was evaluated for its antibacterial activity against Gram-positive and Gram-negative bacteria by MIC determination. The MIC values ranged vastly from 0.6 μg/mL to 5000 μg/mL. In another report, the antibacterial activity of four plant extracts from traditional Chinese medicinal plants (*Agrimonia pilosa* Ledeb, *Iris domestica* (L.) Goldblatt and Mabb, *Anemone chinensis* Bunge, and *Smilax glabra* Roxb) was tested against *Listeria monocytogenes*, *Escherichia coli*, and *Salmonella enterica* [32]. MIC values ranging from 7.81 to 125 μg/mL were obtained. Taking into account the antimicrobial activity of extracts from plants other than pteridophytes, DEE shows a moderate level. However, as reported by Bussmann et al. [33], ethanol extracts showed stronger activity and a much broader spectrum of action than water extracts. For this reason, it is necessary to determine the activity of e.g., ethanolic and methanolic extracts of *D. erythrosora*, as they may show higher antimicrobial activity than the aqueous solution of the ethanolic extract used in the present study.

### 2.3. Effect of DEE Against Human Cancer Cell Lines

**Figure 4:**
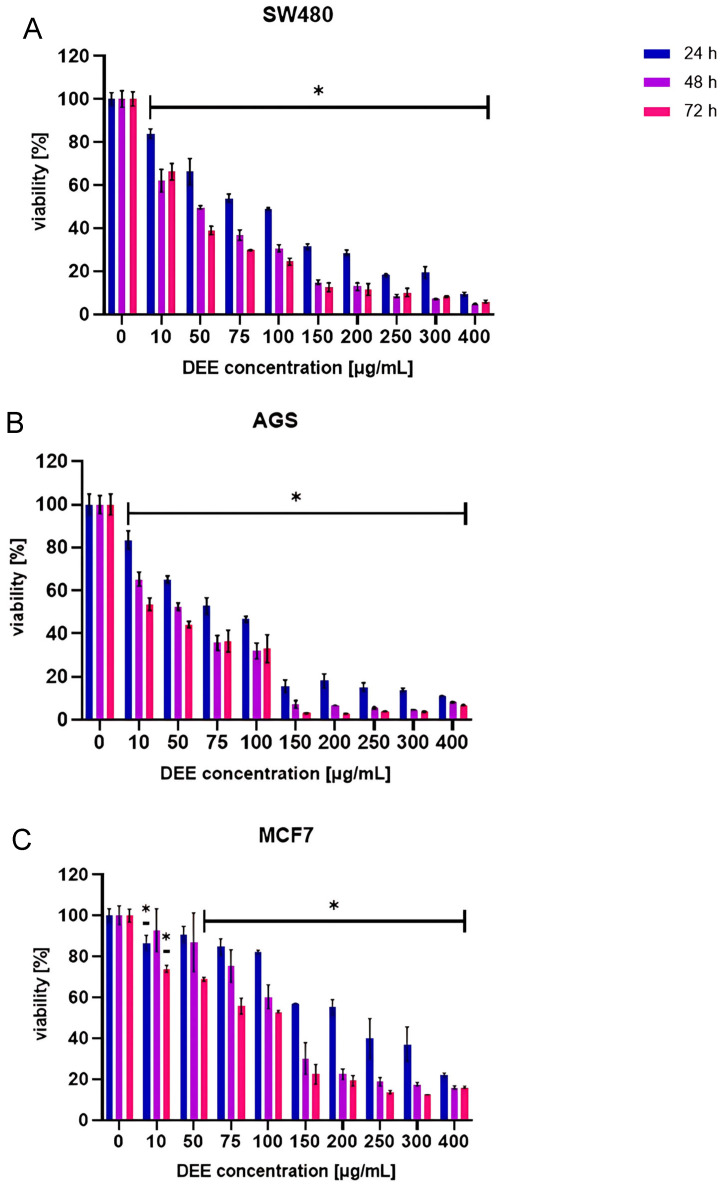
Effect of DEE on the viability (%) of different cancer cell lines: (**A**) SW-480, (**B**) AGS and (**C**) MCF-7 at concentrations of 10–400 µg/mL after 24, 48, and 72 h of incubation, assessed using the MTT assay. Results are presented as means ± SD; data passed the Shapiro–Wilk normality test; one-way ANOVA, n = 3; *p* < 0.05 (*) versus negative control; Dunnett’s test.

In recent decades, scientists pay particular attention to the anticancer activity of plant-derived compounds, especially polyphenols. In order to assess the potential usefulness of the tested extract in anticancer action, its in vitro anticancer activity was studied using cell lines (SW-480, MCF7, AGS) cultured in the presence of the extracts for 24, 48, and 72 h. The MTT (3-[4,5-dimethylthiazol-2-yl]-2,5 diphenyl tetrazolium bromide) assay was used to determine cell viability under the influence of the extract solution. The percentage of cell viability is summarized in Figure 4. The results indicate the influence of the extract on the tested cell lines in a manner dependent on the applied dose and exposure time. The most sensitive of the tested samples were the AGS and SW-480 cell lines. A marked reduction in AGS cell viability was observed at a concentration of 150 µg/mL, with viability decreasing to approximately 15% after 24 h of treatment. Continued exposure for 48 and 72 h at the same concentration further reduced cell viability to below 10%. In the case of the SW-480 cell line, 24 h of treatment resulted in a decrease in viability to below 20% at a concentration of 250 µg/mL. After 48 and 72 h of treatment at the same concentration, cell viability dropped to below 10%.

**Figure 5:**
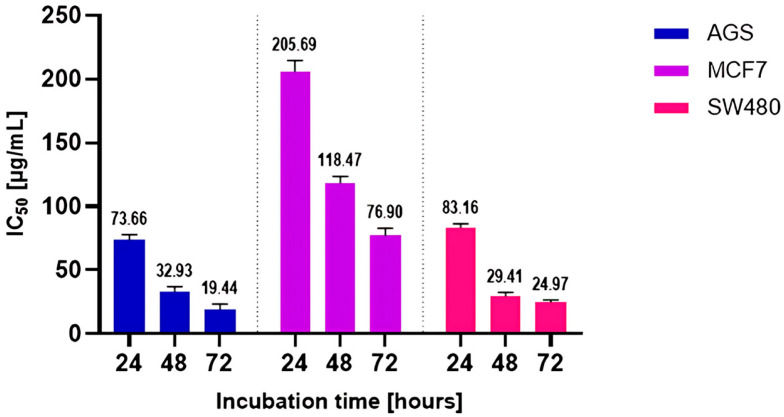
IC_50_ values of DDE against cancer cell lines (AGS, MCF7, SW-480) obtained via the MTT cell viability assay. Data are presented as means ± SEM.

Taking into account the 24 h incubation time, the lowest IC50 value was obtained in the AGS line, while the MCF line was the most resistant to the extract. The values were 73.66 and 205.69 µg/mL, respectively. The other IC50 values for the tested cell lines are summarized in the chart below (Figure 5). Considering the natural origin and complexity of the tested agent, the obtained values can be considered to indicate high in vitro anticancer activity.

The anticancer properties of *D. erythrosora* were also studied by Cao et al. [10]. The results of their research showed that a flavonoid extract from *D. erythrosora* had cytotoxic effects on the carcinomic human alveolar basal epithelial cell line A549. The cell inhibition was estimated at 41.62% at the concentration of 3.6 mg/mL flavonoid extract. Other species of the genus Dryopteris also show anticancer properties. For example, a *D. ramosa* extract exhibited anticancer activity against HepG-2 cells, with an IC_50_ value of 85.67 µg/mL [34]. Both the present study and the research mentioned above reveal the significant potential of *D. erythrosora* as a source of anticancer substances that can be used in anticancer action.

### 2.4. Cytotoxicity and Antiviral Activity of DEE

**Figure 6:**
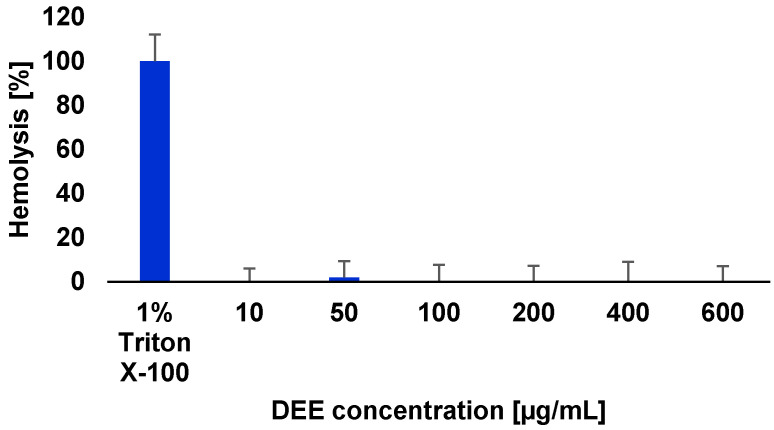
Effect of DEE on sheep erythrocytes. Data are expressed as the mean ± SD of three independent experiments.

Before the antiviral activity of DEE was studied, its cytotoxicity against erythrocytes and VERO cells was determined. In the first step, a hemolytic activity assay was performed on sheep erythrocytes. Triton X-100 and PBS served as positive and negative controls, respectively. The erythrocytes were incubated in the presence of various concentrations of the extract (10–600 µg/mL), and the degree of hemolysis was subsequently assessed. The results indicated that, up to the highest DEE concentration, the level of erythrocyte hemolysis did not exceed 2% (1.95% hemolysis at 50 µg/mL of DEE) (Figure 6). The tested concentrations comprise both the IC_50_ values against the tested cancer cell lines and the MIC values for the most sensitive microorganism, *S. aureus*. These findings suggest that DEE is safe for erythrocytes at doses effective against cancer cells and *S. aureus*.

**Figure 7:**
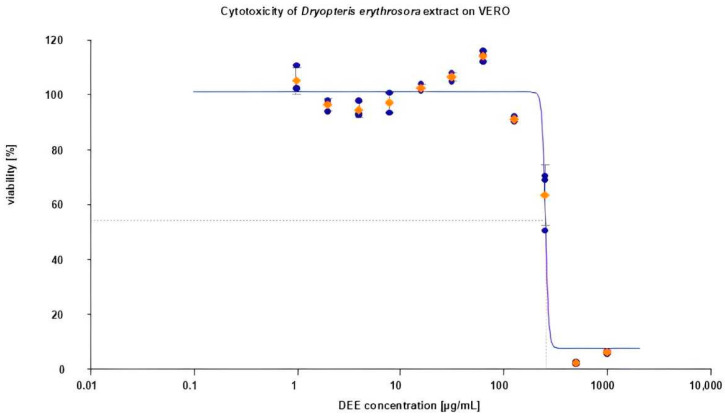
Cytotoxicity of DEE on VERO cells after 72 h incubation.

The extract showed no effect on VERO cells up to a concentration of 250 μg/mL (Figure 7). This value was even 10-fold higher than the IC_50_ of DEE determined against cancer cells, which proves the safety of DEE towards normal cells when tested in anticancer doses.

**Figure 8:**
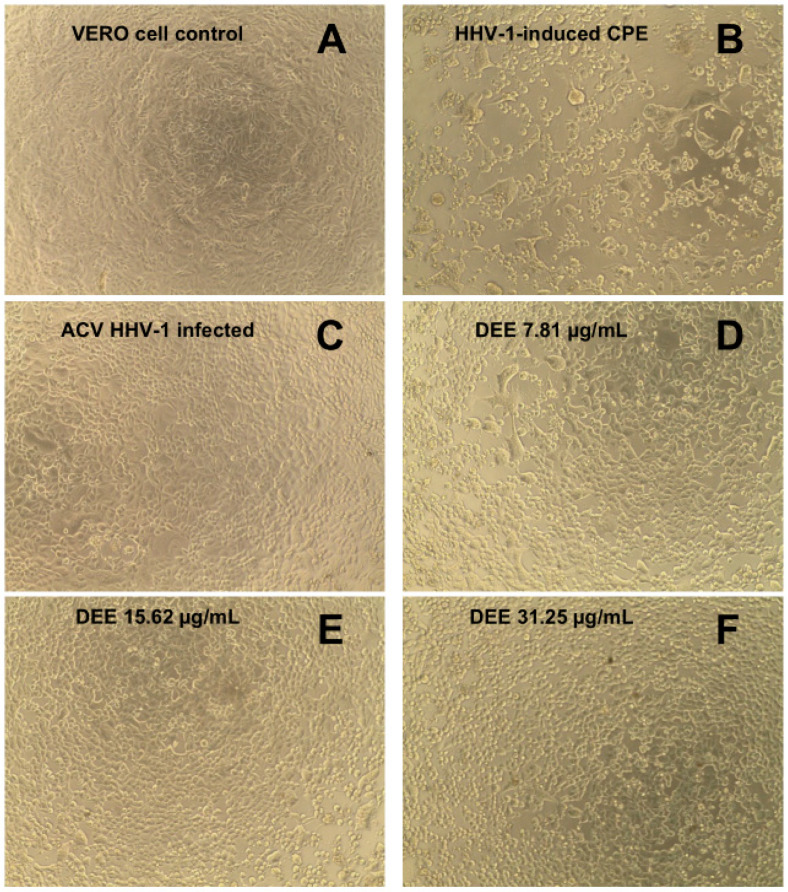
Impact of the extract on the HHV-1-induced cytopathic effect in VERO cells: (**A**)—control cells; (**B**)—HHV-1-induced cytopathic effect, virus control; (**C**)—infected cells treated with acyclovir (60 μg/mL); (**D**–**F**)—infected cells treated with extracts at 7.81, 15.62, and 31.25 μg/mL, respectively.

Antiviral activity was assessed against human herpesvirus type 1 (HHV-1) replicating in VERO cells. The incubation of the HHV-1-infected VERO cells with the extract resulted in a visible reduction in the virus-induced cytopathic effect (CPE), compared to the viral control. An effect on CPE inhibition was observed in samples exposed to extract concentrations of 15.62 and 31.25 μg/mL (Figure 8). Below these values, no inhibition of CPE was observed. Following the CPE assays, the samples were collected and subsequently examined to determine whether the viral load and HHV-1 infectious titer had de-creased.

The determination of CCID_50_ with the viral titer reduction method showed that, at concentrations of 15.62 and 31.25 µg/mL, the extracts affected HHV-1 replication, reducing the level of viral replication by 1.76 log and 1.6 log (Table 5). It can therefore be concluded that the extracts do not show significant antiviral potential since a reduction of the infectious titer by at least 3 log is required.

**Figure 9:**
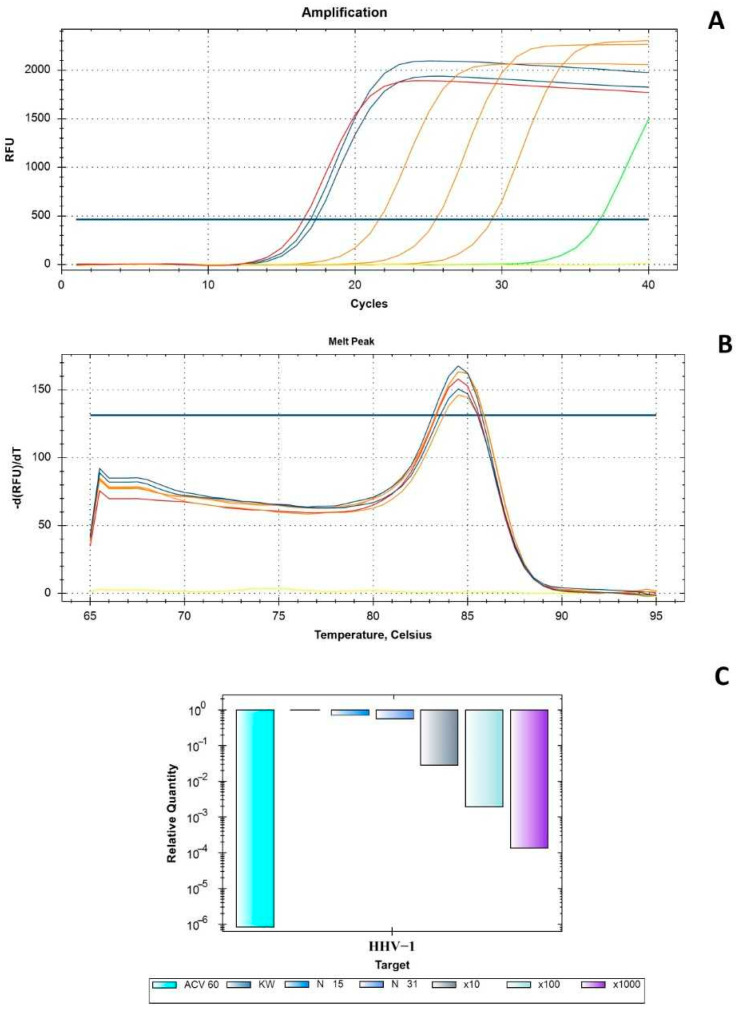
Real-time PCR amplification curves used to measure the HHV-1 virus load ((**A**)—amplification curves; (**B**)—relative quantification; (**C**)—melt curve analysis).

The amplification curve from the real-time PCR analysis of DNA isolates from the DEE-treated samples compared with the viral control (VC) and its dilutions (10×, 100×, and 1000×, respectively), acyclovir (ACV) (60 μg/mL), and the negative control (NC) is shown in Figure 9A. The melting curve analysis (Figure 9B) performed after real-time PCR amplification showed a single peak at 84.5 °C for all the tested samples. Therefore, the same amplicon was found in all the samples, and no primer dimers were observed. The subsequent relative quantification (DCq) performed using CFX Manager Dx software (version 3.1, Bio-Rad Laboratories) (Figure 9C) showed that the tested extracts did not significantly reduce the viral load. Acyclovir (60 μg/mL), used as the reference antiviral drug, prevented the development of CPE in the HHV-1-infected cells and the production of infectious viral progeny. Considering the presented results, it can be concluded that DEE did not show significant antiviral activity. However, it should be noted that this is the first time that the anti-herpesviral activity of DEE has been studied. To date, antiviral studies in the Dryopteris genus have focused on a few species. For example, one study testing the antiviral activity of isolates from Dryopteris atrata rhizomes showed significant antiviral activity against RSV, HSV-1, and H1N1 [35]. Another study showed the activity of *Dryopteris crassirhizoma* extracts against dengue (DENV) and all its serotypes. Extracts from these plants have been found to contain various flavonoids, and some of these compounds’ flavonoids have exhibited potential against dengue in vitro [36].

## 3. Materials and Methods

### 3.1. Preparation of Dryopteris erythrosora Extract (DEE)

**Figure 10:**
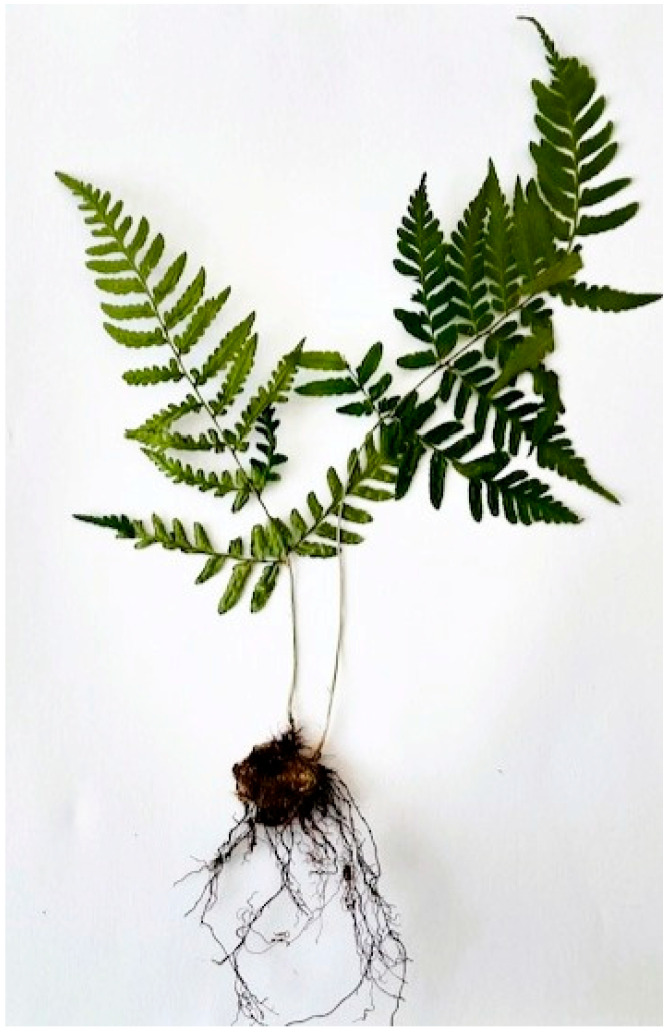
Dried fragment of *D. erythrosora* (herbarium specimen).

*D. erythrosora* plants were purchased from Perfekt Klik Sylwia Grzelak (Ostrzeszow, Poland) and the plants received an EU plant passport (A. Dryopteris B. PL-30/18/20385 C.1/25 D.PL). The aerial parts of the plant were collected and subsequently dried at 37 °C in a thermostatically controlled room. Figure 10 shows a dried fragment of *D. erythrosora* (herbarium specimen). After drying, the plant material was ground into a fine powder using an electric grinder. The extract was obtained using the ethanol extraction method described by Wu et al. [37], with slight modifications. In this method, 20 g of a dried and crushed plant were weighed, transferred to a flask, and poured with 200 mL of 50% ethanol. The sample was extracted in an ultrasonic bath at 60 °C for 30 min. Next, the extract was filtered and collected, the remaining material was poured again with 200 mL of 50% ethanol, and the extraction was repeated. The extract was filtered and added to its previous portion. The residual material was then poured with 200 mL of 80% ethanol and extracted again in an ultrasonic bath (60 °C, 30 min). This step was repeated twice. After completing the extraction process, all the extracts were combined. Subsequently, the extracts were dried using a rotary evaporator (Heidolph Hei-VAP Core) and freeze-dried using a lyophilizer (Christ Alpha 1-2 LD plus). From 1 g of dry plant material, 74 mg of dry extract was obtained. In order to examine its biological properties, the extract was dissolved in water and further analyzed.

### 3.2. Total Phenolic Content (TPC)

The TPC was assayed with the method described in detail by Olech et al. [38]. For this purpose, the Folin–Ciocalteu reagent method was used, and the test was performed on a microtiter plate. Briefly, 20 µL of Folin–Ciocalteu reagent (diluted 1:4 (*v*/*v*) in water), 160 µL of sodium carbonate (75 g/L), and the tested extract in various concentrations (from 1 to 10 mg/mL) were added to the plate wells. At the same time, the test was performed using a standard solution of gallic acid at a concentration of 25–150 µg/mL. Three replicates were performed for each sample. The plate was successively incubated for 20 min at room temperature. After this time, absorbance was measured at 760 nm using a BioTek Synergy H1 microplate reader (BioTek, Winooski, VT, USA) with Gen5 3.10 software (BioTek, Winooski, VT, USA). Based on the results obtained for the gallic acid solution, a calibration curve was plotted, on the basis of which the result was calculated and expressed in mg of gallic acid equivalent per 1 g of DEE.

### 3.3. Total Flavonoid Content (TFC)

The TFC was assayed with the Lamaison and Carnart method as modified and described by Olech et al. [38]. For this purpose, 20 µL of the extract, 160 µL of methanol, and 20 µL of a 2% AlCl_3_ solution were mixed in the wells of a 96-well microtiter plate. At the same time, the test was performed using a standard solution of quercetin at a concentration of 62.5–1000 µg/mL. Methanol was used as a blank. All samples were prepared in triplicate. The plate was successively incubated for 30 min at 28 °C with shaking. After incubation, the absorbance of the samples was measured at 430 nm using a BioTek Synergy H1 microplate reader. Based on the results obtained for the quercetin solution, a calibration curve was plotted, on the basis of which the result was calculated and expressed in mg of quercetin equivalent per 1 g of DEE.

### 3.4. Ferric Reducing Antioxidant Power (FRAP) Assay

The FRAP test was performed using a kit in accordance with the manufacturer’s instructions (Sigma-Aldrich). Briefly, samples of the control, background, and DEE (10 µL) were placed in the wells of a 96-well microtiter plate. Quercetin was used as a reference sample. Next, 190 µL of the Reaction Mix were added successively to the wells. The absorbance of the samples was then measured at 594 nm in the kinetic mode for 60 min at 37 °C using a BioTek Synergy H1 microplate reader.

### 3.5. Antibacterial and Antifungal Activity

Antibacterial properties were determined against reference strains of Gram-positive (*Staphylococcus aureus* (PCM2101), *Staphylococcus epidermidis* (PCM2128)) and Gram-negative (*Escherichia coli* (ATCC8739), *Klebsiella pneumoniae* (PCM2070)) bacteria. Antifungal properties were tested using a *Candida albicans* strain (ATCC10231). The properties were determined with broth microdilution methods in 96-well plates, with determination of MICs (minimum inhibitory concentrations). MIC values were determined using the microdilution method according to CLSI [39] and as described by Khabnadideh et al. [40], with some modifications. Before the test, the microorganisms were cultured on a dedicated medium (nutrient agar for bacteria and Sabouraud agar for fungi). Colonies of the microorganisms were transferred from Petri dishes to 5 mL of sterile 0.85% NaCl, and turbidity was adjusted to 0.5 McFarland. Next, the suspension was diluted 1:200 in RPMI-1640 medium for *C. albicans* and in Mueller–Hinton broth for the bacterial strains. Two-fold serial dilutions of DEE were prepared using microtiter plates. Then, 20 µL of the inoculum was added to the wells. The final volume per well was 100 µL. The trays were incubated at 37 °C for 48 h. Additionally, 100 µL of uninoculated medium was used as a sterility control (blank). The MIC was taken as the lowest concentration of the tested compound that inhibits visible microorganism growth. The test was performed in triplicate.

### 3.6. Anticancer Activity

AGS (gastric adenocarcinoma), MCF7 (breast adenocarcinoma), and SW480 (colorectal adenocarcinoma) (ATCC) cell lines were cultivated before the test as described elsewhere. Briefly, the cells were grown in Dulbecco’s Modification of Eagle’s Medium (DMEM) containing glucose (4.5 g/L), sodium pyruvate, L-glutamine, 10% fetal bovine serum (FBS), and 1% antibiotics (100 U/mL penicillin and 1000 µg/mL streptomycin). The cells were grown in an incubator in a humidified atmosphere of 95% air and 5% carbon dioxide and at 37 °C. The cells, at a density of 1.5 × 10^4^ cells/well, were treated with increasing DEE concentrations (10–400 µg/mL) for 24, 48, and 72 h. Cell viability was assessed using MTT assays according to the standard protocol described in the literature. Briefly, after the incubation period, MTT reagent was added to each well. Next, the plates were incubated in a humidified atmosphere (95% air and 5% carbon dioxide and at 37 °C) for 3 h. After incubation, DMSO was added into each well, and the plate was shaken on an orbital shaker. Finally, measurement of absorbance at 570 nm was carried out using a Synergy H1 microplate reader.

### 3.7. Hemolytic Activity

Sheep erythrocytes were collected by centrifuging blood samples (2000 rpm) for 10 min at 20 °C and sequentially washed three times with a PBS solution. After washing, the erythrocytes were suspended in the PBS buffer at a final concentration of 2%. At the same time, appropriate concentrations of the tested extracts (0–600 µg/mL) were prepared in a final volume of 50 µL. A 1% Triton X-100 solution was used as an example of complete hemolysis. The tested extracts were mixed with 450 µL of 2% erythrocyte suspension. The samples were incubated for 1 h at 37 °C and then centrifuged at 2000 rpm (10 min, 20 °C). Then, 150 μL of the supernatant was transferred to the wells of a microtiter plate, and absorbance was sequentially measured on an automatic plate reader (Synergy H1) at 450 nm. The test was performed in triplicate.

### 3.8. Cytotoxicity and Antiviral Properties

VERO cells were grown in Dulbecco Modified Eagle’s Medium (Corning, Tewksbury, MA, USA), which was supplemented with fetal bovine serum (FBS; Capricorn Scientific, Ebsdorfergrund, Germany) and antibiotics (Corning’s penicillin-streptomycin solution). Trypsin and phosphate-buffered saline (PBS) were purchased from Corning, while dimethylsulfoxide and 3-(4,5-dimethylthiazol-2-yl)-2,5-diphenyltetrazolium bromide (MTT) were acquired from Sigma-Aldrich (St. Louis, MO, USA). The investigation was conducted using a CO_2_ incubator (Panasonic Healthcare Co., Ltd., Tokyo, Japan) at 37 °C with 5% CO_2_. The final point dilution test method was used to evaluate infectious titers (CCID_50_—50% of the infectious cell culture dose) after HHV-1 (ATCC, No. VR-260) had propagated in the VERO cells.

#### 3.8.1. Cytotoxicity Testing

The previously outlined protocol was used when testing for cytotoxicity [41]. Serial dilutions (2000–0.98 µg/mL) of the extracts in cell culture media were applied to VERO cell monolayers in 96-well plates for 72 h. Then, the media were removed. Following a PBS wash, an MTT solution in DMEM was added, and the cells were incubated for another four hours. Finally, the SDS/DMF/PBS solvent was used to dissolve formazan crystals. The absorbance (540 and 620 nm) was measured using a Synergy H1 Multi-Mode Microplate Reader (BioTek Instruments, Inc., Winooski, VT, USA) following an overnight incubation period. The data were exported to GraphPad Prism (version 7.0.4) for additional analysis by Gen5 software (version 3.09.07; BioTek Instruments, Inc., Winooski, VT, USA).

#### 3.8.2. Antiviral Activity

Antiviral activity was examined in accordance with previous literature [41]. HHV-1 was introduced into VERO cells grown in 96-well plates at 100-fold infectious titers of CCID_50_. Following a one-hour pre-incubation period to facilitate virus attachment, the PBS-washed cell monolayer was subjected to extract treatment at non-toxic concentrations (250–0.49 μg/mL). Based on the dose–response curves acquired during the cytotoxicity investigations, non-toxic doses were selected. The virus control (infected, untreated) cells were incubated further until a typical cytopathic effect (CPE) was observed. Then, using an inverted microscope (CKX41, Olympus Corporation, Tokyo, Japan) fitted with a camera (Moticam 3+, Motic, Hong Kong, China), the extract-treated infected cells were also examined for CPE. The outcomes were then recorded (Motic Images Plus 2.0, Motic, Hong Kong, China). Samples were taken and stored frozen at −72 °C until the infectious virus titer was determined. The plates were then frozen (−72 °C) and thawed three times. A final dilution assay was used to carry out the virus titration. In summary, VERO monolayers in 96-well plates were subjected to samples from the previously disclosed antiviral experiments at 10-fold dilutions. Using the MTT test, virus titers were determined after 72 h. Antiviral activity was determined by comparing the differences in the virus titer between the extract-treated samples and the control virus sample. A decrease of at least three logs in the infectious virus titer is required for significant antiviral activity.

Following the manufacturer’s instructions, the viral DNA was isolated using a commercially available kit (QIAamp DNA Mini Kit, QIAGEN GmbH, Hilden, Germany). Using primers (UL54F—5′ CGCCAAGAAAATTTCATCGAG 3′ and UL54R—5′ ACATCTTGCACCACGCCAG 3′) and SybrAdvantage qPCR Premix (Takara Bio Inc., Kusatsu, Shiga Prefecture, Japan), real-time PCR analysis was performed on a CFX96 thermal cycler (Bio-Rad Laboratories, Inc., Hercules, CA, USA). Using CFX ManagerTM Dx Software (version 3.1, Bio-Rad Laboratories, Inc., Hercules, CA, USA), the viral load of HHV-1 in the extract-treated samples was evaluated in relation to the virus control based on the relative quantity (DCq) method.

## 4. Conclusions

Currently, there are relatively few data in the literature regarding the bioactivity of ferns despite their potential as sources of new bioactive compounds. Further analysis and testing of wild fern species to determine their biological properties may result in finding potential compounds with excellent antibacterial or anticancer properties that could improve the arsenal of agents used to treat various bacterial infections, cancers, and perhaps even other oxidative stress-related diseases. Moreover, the present results may serve as a basis for further research on the potential use of *Dryopteris erythrosora* in phytotherapy, dietary supplements, cosmetics, and nutraceuticals. To our knowledge, this report shows a pioneering study of the biological properties of *D. erythrosora*. Among the three biological activities of the DEE tested here, the anticancer effect appears to be the most pronounced and worth further exploration. Moreover, DEE shows a high level of safety against erythrocytes and monkey kidney cells, which reveals its capability of discrimination between normal and cancer cells. It should be noted, however, that this study was conducted using a crude ethanol extract, which was subsequently dissolved in water. Therefore, the low biological activity observed in this study may result from the presence of a mixture of compounds with varying polarity, not all of which exhibit biological effects or may even counteract each other’s activity. Future in-depth phytochemical and molecular biology analyses of DEE and its components should disclose compounds responsible for this property as well as their mechanism of action in cancer cells. In spite of the low antimicrobial and antiviral potential of DEE, these assays should be reconducted with the compounds separated from the DEE using different solvents. Further studies on the safety of the investigated extract are also necessary, including its potential toxicity during long-term use and its effects on various cell types and tissues.

## Figures and Tables

**Table 1:** Total content of polyphenols and flavonoids in plants of the genus *Dryopteris* and in other ferns and higher plants.

Species	Solvent Used for Extraction	TPC	TFC	Reference
*Dryopteris juxtapostia*	methanol; dichloromethan	163.00 mg GAE/g; 222.00 mg GAE/g;	83.7 mg QE/g; 51 mg QE/g	[18]
*Dryopteris ramosa*	ethyl acetate; methanol; water	-	45.28 μg QE/mg; 36.94 μg QE/mg; 25.69 μgQE/mg	[20]
*Dryopteris ramosa*	methanol; dichloromethan	199.20 ± 4.50 mg GAE/g; 184.20 ± 4.04 mg GAE/g	50.13 ± 3.51 mg RE/g; 73.02 ± 1.00 mg RE/g;	[21]
*Dryopteris crassirhizoma*	ethanol	-	208.09 ± 5.89 mg RE/g	[22]
*Dryopteris affinis*	methanol	112.54 ± 8.09 mg GAE/g	-	[23]
*Polystichum woronowii*	methanol	180.00 ± 12.5 mg GAE/g	-	[23]
*Pteris cretica*	methanol	71.86 ± 5.34 mg GAE/g	-	[23]
*Asplenium adiantum*	methanol	21.85 ± 3.12 mg GAE/g	-	[23]
*Basella alba*	methanol	72.66 ± 0.46 mg GAE/g	6.97 ± 0.62 mg QE/g	[18]
*Ipomoea aquatica*	methanol	77.06 ± 0.70 mg GAE/g	6.61 ± 0.42 mg QE/g	[18]
*Solanum nigrum*	methanol	97.96 ± 0.62 mg GAE/g	16.42 ± 0.39 mg QE/g	[18]
*Digera muricata*	methanol	83.69 ± 0.46 mg GAE/g	18.00 ± 0.68 mg QE/g	[18]
*Cassia tora*	methanol	287.73 ± 0.16 mg GAE/g	37.86 ± 0.53 mg QE/g	[18]
*Portulaca oleracea*	methanol	216.96 ± 0.87 mg GAE/g	39.38 ± 0.57 mg QE/g	[18]

**Table 2:** MIC values (mg/mL) of DEE against bacteria and fungus.

Microorganisms	MIC (mg/mL)
*Escherichia coli*	1.25
*Klebsiella pneumoniae*	0.375
*Staphylococcus aureus*	0.375
*Staphylococcus epidermidis*	0.75
*Candida albicans*	>10

**Table 3:** Comparison of antimicrobial activity classifications based on MIC values.

MIC Range (µg/mL)	Kuete and Efferth (2010) Classification [28]	Silva et al. (2013) Classification [29]
<100	Significant activity	Highly active
100–500	Moderate activity	Active
501–625	Moderate activity	Moderately active
626–1000	Weak activity	Moderately active
1001–2000	–	Weakly active
>2000	–	Inactive

**Table 4:** MIC of selected medicinal plants traditionally used in northern Peru against *Escherichia coli* and *Staphylococcus aureus*, based on data from Bussmann et al. [30].

Tested Plant	MIC of the Ethanol Extract Against *E. coli* [mg/mL]	MIC of the Ethanol Extract Against *S. aureus* [mg/mL]
*Iresine herbstii* Hook.	256	-
*Apium graveolens* L.	32	256
*Vallesia glabra* (Cav.) Link	64	16
*Baccharis* sp	2	4
*Senecio* sp.	8	2
*Ochroma pyramidale* (Cav. ex Lam.) Urb.	1	-
*Senna bicapsularis* (L.) Roxb.	0.016	256
*Banisteriopsis caapi* (Spruce ex Grieseb.) Morton	0.0625	1

**Table 5:** Antiviral activity of DEE against HHV-1.

Compounds	Concentration (µg/mL)	(CCID_50_/mL) ^a^
DEE	15.62	4.09 ± 0.51
	31.25	4.25 ± 0.30
Acyclovir	60	0
Virus control	−	5.85 ± 0.03

^a^ The virus titers are shown in a log scale. Data represent the mean ± SD of 3 independent experiments.

## Data Availability

The data presented in this study are available on request from the corresponding author.
